# An interpretable radiomics–deep learning nomogram from whole‐body bone scintigraphy for MDP‐avid bone metastasis prediction in NSCLC

**DOI:** 10.1002/acm2.70730

**Published:** 2026-08-03

**Authors:** Weihao Zhai, Xiaolin Li, Qian Zhou, Taohu Zhou, Yi Wang, Xiuxiu Zhou, Xin'ang Jiang, Ziwei Zhang, Qianxi Jin, Shiyuan Liu, Li Fan

**Affiliations:** ^1^ Department of Radiology Second Affiliated Hospital of Naval Medical University Shanghai China; ^2^ College of Health Sciences and Engineering University of Shanghai for Science and Technology Shanghai China; ^3^ Department of Nuclear Medicine Huadong Hospital Affiliated to Fudan University Shanghai China

**Keywords:** bone metastasis, deep learning, non‐small cell lung cancer, radiomics, radionuclide imaging

## Abstract

**Background:**

Whole‐body bone scintigraphy (WBS) remains a widely used first‐line screening tool for bone metastasis, but differentiating MDP‐avid metastatic lesions from benign bone abnormalities in non‐small cell lung cancer (NSCLC) remains challenging.

**Purpose:**

To develop and externally validate a radiomics and deep learning bone signature (RDB) derived from planar WBS images for prediction of MDP‐avid bone metastasis and to explore its prognostic value in NSCLC.

**Methods:**

A retrospective analysis was conducted on NSCLC patients who underwent WBS imaging across two centers. Eight hundred twenty‐nine patients from Center 1 were used for training and internal validation, whereas 574 patients from Center 2 were used for external validation. Radiomics features from 2 segmentation methods and deep learning features were extracted from planar WBS images. The RDB nomogram was generated from clinical, radiomics, and deep learning features using the best‐performing machine learning algorithm. Model performance was assessed using the area under the receiver operating characteristic curve. Shapley additive explanations were used to interpret the model output, and Kaplan‐Meier survival analysis evaluated prognostic stratification. The locked model was also translated into a research‐use‐only local desktop application.

**Results:**

There were 720 patients with bone metastasis (202 solitary and 518 multiple) and 683 without bone metastasis. The final RDB nomogram included 8 radiomics features, 11 deep learning features, and 2 clinical factors. The RDB nomogram achieved an area under the curve of 0.857 (95% CI, 0.811–0.904) in the internal validation cohort and 0.870 (95% CI, 0.840–0.900) in the external validation cohort for predicting MDP‐avid bone metastasis. The Rad‐score was the most influential predictor. Kaplan‐Meier analysis showed significant survival differences for solitary‐lesion patients in both centers. A deployable desktop implementation of the final model was completed to support reproducibility and external use.

**Conclusions:**

The RDB nomogram is a promising noninvasive tool for predicting MDP‐avid bone metastasis in NSCLC patients and may provide prognostic value, particularly for solitary lesions.

## 1 INTRODUCTION

Lung cancer is the leading cause of cancer‐related mortality worldwide, accounting for approximately 20% of cancer deaths.^1^, ^2^ Non‐small cell lung cancer (NSCLC) is the most common type, accounting for almost 80% of all lung cancer subtypes.^3^

NSCLC is characterized by a high prevalence of bone metastasis (BM), with up to 40% of patients developing BM.^4^ BM in NSCLC manifests in three primary forms: osteolytic (bone destruction), osteoblastic (bone formation), or mixed osteolytic‐osteoblastic lesions, each driven by distinct molecular mechanisms of tumor‐bone interactions.^5^, ^6^ Osteoblastic metastases, characterized by excessive osteoblast activation and sclerotic bone formation, are particularly common in NSCLC and exhibit MDP‐avid (defined as lesions with intense 99mTc‐MDP uptake) characteristic on planar whole body bone scintigraphy (WBS) due to heightened osteoblastic activity.^7^, ^8^ These lesions appear as single or multiple “hot spots” on WBS, reflecting localized bone remodeling. While WBS is highly sensitive for detecting osteoblastic change due to its ability to capture increased radionuclide concentration, pathologically it might be early‐stage BM or benign conditions such as degenerative changes, trauma, or infection.^9^, ^10^ Accurate differentiation of these lesions is crucial, as misdiagnosis may result in delayed treatment or unnecessary invasive interventions.

Single Photon Emission Computed Tomography/Computed Tomography (SPECT/CT) has emerged as a method to improve specificity, yet it shares limitations with WBS in distinguishing MDP‐avid BM from benign lesions.^11^ A study by Yang et al. demonstrated that SPECT/CT, while improving specificity compared to planar WBS, still exhibits a false‐positive rate of up to 25% in solitary lesions due to overlapping imaging features between inflammatory and metastatic processes.^12^ Helyar et al. further emphasized this challenge, reporting that SPECT/CT misclassified 18% of solitary MDP‐avid lesions in NSCLC patients, primarily due to mimicking features of benign bone remodeling or infection.^13^ Computed tomography (CT), the gold standard for identifying osteolytic lesions through its high spatial resolution and ability to visualize bone destruction, is less effective for early osteoblastic BM, as structural changes may be subtle or absent in initial stages.^14^, ^15^ Furthermore, CT often lags behind WBS in detecting osteoblastic BM, as morphological alterations may take weeks to months to manifest despite active metastatic progression.^16^

Although there are still many gamma cameras and SPECT in use, and planar WBS still remains valuable as the first‐choice screening modality due to its low cost, low radiation dose, and whole‐body skeleton visualization. Thus, there is an urgent need for innovative approaches to enhance MDP‐avid BM prediction in WBS images, preserving its role as an effective initial screening tool.

Radiomics is an effective method for quantitatively analyzing medical images by extracting high‐throughput features from lesions.^17^, ^18^ Machine learning (ML) can be combined with radiomics features to develop models for a specific diagnosis.^19^ The deep learning (DL) methodology, which utilizes convolutional neural networks, can also learn complex features from images and has demonstrated outstanding results in many classification tasks.^20^ Previous bone scintigraphy and SPECT/CT radiomics studies have focused on recurrence prediction in prostate cancer or differentiation of bone metastases from benign bone lesions, often with smaller cohorts, lesion‐level SPECT/CT features, or limited external validation.21–25 However, few studies have integrated planar WBS radiomics and DL features to predict MDP‐avid BM in NSCLC and assess prognostic value. The aim of this work was to develop a radiomics and DL bone signature (RDB) based on WBS to classify MDP‐avid BM and explore its prognostic value.

## 2 METHODS

### 2.1 Patients

**FIGURE 1:**
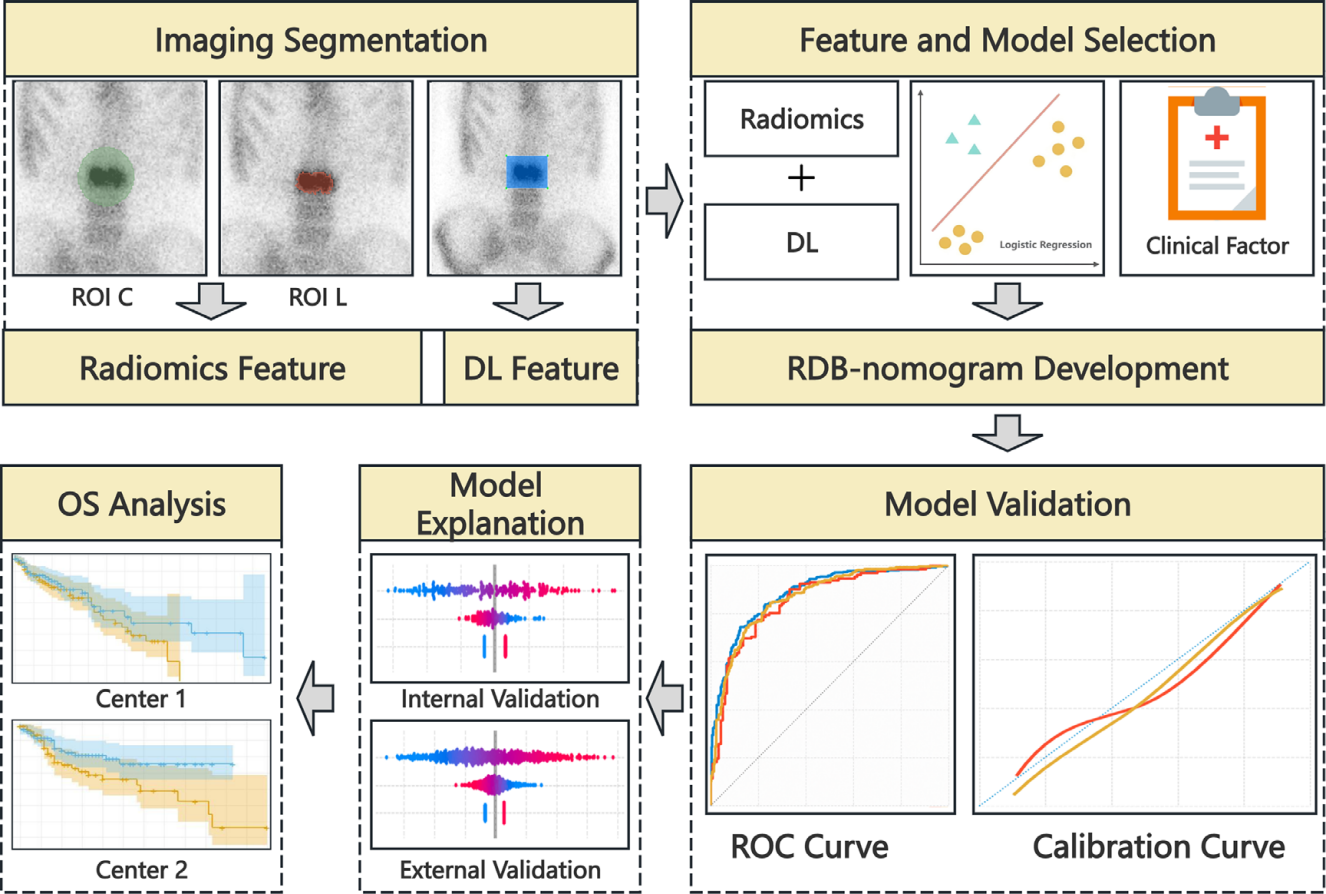
The flowchart of the study.

This retrospective study was approved by the institutional review boards at both participating centers, and the requirement for informed consent was waived because of the retrospective design (No.2024SL135). Figure 1 summarizes the overall workflow, including patient selection, image segmentation, radiomics and DL feature extraction, feature and model selection, model validation, SHAP interpretation, OS analysis, and software implementation.

We retrospectively reviewed data from patients with NSCLC who underwent WBS imaging after the first diagnosis across 2 centers between January 2016 and March 2024. The training and internal validation cohorts were derived from Center 1, whereas the external validation cohort was obtained from Center 2.

The inclusion criteria were as follows: (1) patients with a history of NSCLC; (2) WBS images displaying abnormal MDP‐avid bone lesion. The exclusion criteria included: (1) abnormal bone lesions without a definite diagnosis; (2) previous history of other malignant tumors; and (3) loss of clinical information or poor image quality. The definition of BM was confirmed based on the following methods: (1) the puncturing or pathological results showed BM; (2) at least one of the other imaging (SPECT/CT, CT, MRI) findings indicated BM after a minimum of six months of follow‐up. The clinical information was obtained from medical records or by telephone interview.

The endpoints of this study were overall survival (OS). OS was defined as the duration from the identification of BM on the WBS image to the date of death from any cause, or was censored at the last known contact if the patient remained alive. For the OS analysis, only BM patients with confirmable vital status and survival time from medical records or telephone follow‐up were included; patients without reliable OS information were excluded from survival analysis. The follow‐up period for all patients continued until August 2024.

### 2.2 Image acquisition

Planar WBS was performed in both centers using a Philips BrightView XCT dual‐head SPECT/CT, with anterior and posterior views acquired 3–4 h after intravenous injection of 925 MBq (25 mCi) 99mTc‐MDP. Low‐energy, high‐resolution parallel‐hole collimators and a 140 keV photo peak with 20% windows were employed at a table speed of 15 cm/min.

### 2.3 Radiomics features extraction

For each patient's imaging, a nuclear medicine physician with 8 years of experience identified the MDP‐avid bone lesion with a definite diagnosis and manually segmented the region of interest (ROI) using 3D Slicer 5.2.2 software, employing two methods illustrated in Figure 1: (1) creating a circle‐shaped ROI that fully encapsulated the lesion (ROIC), with the circle diameter determined by extending 10 mm beyond both sides of the lesion's longest axis; and (2) manually contouring an ROI along the boundaries of the MDP‐avid lesion area (ROIL). Another senior physician with over ten years of experience reviewed the final ROIs. For patients with multiple BM, the most prominent lesion, defined primarily as the lesion with the highest visual MDP uptake and clearest lesion‐to‐background contrast, was chosen for segmentation on the WBS image. When several lesions were comparable, the final lesion was selected by consensus. Any different opinion was settled by consensus.

Radiomics features were extracted respectively from both ROI_C_ and ROI_L_ of original and wavelet‐filtered images using the Pyradiomics package (version 3.0.1), including first‐order features, shape‐based, grey‐level co‐occurrence matrix (GLCM), grey‐level dependence matrix (GLDM), grey‐level run length matrix (GLRLM), grey‐level size zone matrix (GLSZM), and neighboring grey‐tone difference matrix (NGTDM).

The counts information of bone lesions, including Max Counts, Mean Counts, Min Counts, and Standard Deviation (SD) of Counts, was also obtained by the 3D Slicer segmentation module.

### 2.4 Deep learning feature extraction

A pre‐trained ResNet‐101 architecture was employed for the purpose of extracting DL features from the WBS images. A nuclear medicine physician with 12 years experience drew a rectangular ROI that included the entire bone lesion using the labelimg package (version 0.4.3). The dimensions of the rectangular ROI were established by extending 10 mm outward in every direction from the lesion's margins. The WBS images were then resized to a fixed size of 512×512 pixels in order to align with the input requirements of the ResNet‐101 model. Following the removal of the final fully connected layer, DL features were extracted from the last convolutional layer of the model.

### 2.5 Feature selection and model establishment

Patients in Center 1 were randomly split into a training cohort and an internal validation cohort with a ratio of 7:3. All feature selection steps were based on the training cohort. We first conducted the Student *t*‐test to remove features without significant differences between BM and non‐BM lesions and to reduce dimensionality before multivariable modeling; features with an adjusted *p* value below 0.05 were selected and normalized using the z‐score. Then, we employed the least absolute shrinkage and selection operator (LASSO) logistic regression method to select the most predictive features with 10‐fold cross‐validation. Then, the selected ROI_C_, ROI_L_, and DL features were trained with 5 common ML algorithms, including logistic regression, SVC, random forest, KNN, and XGBoost. GridsearchCV was performed to select optimal hyperparameters for each algorithm (File S1). After comparison of the performance of ROI_C_ and ROI_L_ features among 5 ML algorithms, the better radiomics features and deep learning features will undergo feature reselection and develop the combined radiomics and DL bone signature (RDB) model based on the best ML algorithm. A Rad‐score was calculated using a linear combination of selected features weighted by the LASSO algorithm.

### 2.6 Development of the RDB‐nomogram

Statistically significant different clinical factors in predicting BM were evaluated using both univariate and multivariable logistic regression analyses in Center 1. To enhance the visual representation of the model, we have presented it in the form of a nomogram, which consisted of the chosen clinical factors and RDB Rad‐score. The probability of each impact factor can be translated to a score based on the points at the top of the nomogram. The risk is calculated by adding the scores of each impact factor corresponding to the bottom of the nomogram.

### 2.7 Segmentation reproducibility analysis

A randomly selected subset of 50 patients was independently segmented by two nuclear medicine physicians using the ROIC and DL bounding‐box methods. All 8 ROIC radiomics features and 11 DL features included in the final model were extracted from each segmentation. Inter‐observer reproducibility was evaluated using a two‐way random‐effects, absolute‐agreement, single‐measurement ICC with 95% confidence intervals. An ICC ≥0.75 was considered acceptable.

### 2.8 Model explanation

The Shapley Additive Explanations (SHAP) algorithm was employed to elucidate the contribution of each factor to the final prediction output of the RDB‐nomogram in the internal and external cohorts. The SHAP assigned an important value to each factor for every prediction, visualizing the contribution of each item to the model's prediction and highlighting its effect on the likelihood of a specific outcome.^21^, ^22^

### 2.9 The evaluation of prediction for overall survival

To assess the predictive ability of our RDB‐nomogram for OS in NSCLC patients with BM, we conducted follow‐up studies on patients from Center 1 and Center 2, focusing on those with BM. Patients were stratified into high‐risk and low‐risk groups based on the optimal cutoff value determined by the receiver operating characteristic (ROC) curve, which was derived from the total points of the RDB‐nomogram and actual survival status. Kaplan‐Meier (KM) survival analysis was performed to evaluate the differences in survival outcomes between the risk groups in both centers. Subgroup analyses were further conducted to assess the predictive performance of the nomogram in patients with solitary and multiple BM lesions.

### 2.10 Software implementation and open‐source deployment

To facilitate reproducibility and practical translation of the final model, we implemented the locked RDB‐nomogram as a research‐use‐only local desktop application. For researchers, by uploading a single WBS image and completing a manual segmentation, the software will be able to output both BM diagnosis and prognostic risk stratification results.

### 2.11 Statistical analysis

Python (version 3.11), R software (http://www.R‐project.org; Version 4.4.1) and Medcalc software (version 22.001) were employed for statistical analysis. The Shapiro‐Wilk normality test is employed to assess the normality of continuous sample data. If the data conforms to a normal distribution, it is represented by the mean ± standard deviation, and independent one‐way analysis of variance is employed for intergroup comparison. Conversely, if the data does not conform to a normal distribution, the median (25th percentile, 75th percentile) is utilized for representation, and the Kruskal‐Wallis test is applied for intergroup comparison. Qualitative classification data is analyzed using frequency (%), and intergroup comparisons are conducted via the chi‐square test or Fisher's exact test. The ROC curve and decision curve analyses (DCA) were used to evaluate and compare the predictive performance of the models. The AUCs of different models were compared using the Delong test. The Kaplan‐Meier curve and log‐rank test were used to determine the significance of the observed discrepancies.

## 3 RESULTS

### 3.1 Patient characteristics

Between January 2016 and March 2024, a retrospective analysis was conducted on 1,832 and 1,089 patients at Centers 1 and 2. Finally, A total of 1,403 patients (846 males, 557 females) were enrolled, comprising 829 patients (518 males, 311 females) from Center 1 and 574 patients (328 males, 246 females) from Center 2. In Center 1, 441 BM and 388 non‐BM patients were randomly divided into a training and internal validation cohorts. 279 BM and 295 non‐BM patients from Center 2 were set as the external validation cohort. Among all patients with evidence of BM, there were 202 solitary BM patients (135 from Center 1 and 67 from Center 2) and 518 multiple BM patients (306 from Center 1 and 212 from Center 2).The mean age were 63.5 ± 9.9 and 67.3 ± 10.1 in both centers. There were no significant differences in gender, lesion location, or acquisition view among all three cohorts (*p* > 0.05). Table 1 summarizes the three cohorts' patient characteristics.

**TABLE 1:** Patient characteristics.

	Training (*n* = 580)	Internal Validation (*n* = 249)	External Validation (*n* = 574)	*p*
Age	65 (58,71)	63 (57,70)	68 (62,74)	< 0.001
Gender				0.129
Female	219 (37.76%)	92 (36.95%)	246 (42.86%)	
Male	361 (62.24%)	157 (63.05%)	328 (57.14%)	
Location				0.098
Cranium	8 (1.38%)	3 (1.2%)	11 (1.92%)	
Spine	265 (45.69%)	117 (46.99%)	278 (48.43%)	
Rib/Sternum	156 (26.9%)	64 (25.7%)	113 (19.69%)	
Pelvis	74 (12.76%)	41 (16.47%)	94 (16.38%)	
Limb	77 (13.28%)	24 (9.64%)	78 (13.59%)	
Acquisition view				0.188
Anterior	280 (48.28%)	123 (49.4%)	251 (43.73%)	
Posterior	300 (51.72%)	126 (50.6%)	323 (56.27%)	
Lesion voxel number	161 (91,285.25)	153 (95,273)	196 (120,327.5)	< 0.001
Lesion Size(mm^2^)	649.81 (367.28,1151.29)	617.52 (383.43,1101.85)	792.12 (485.55,1321.87)	< 0.001
Counts_max	54 (41,74)	53 (42,75)	66 (52,96)	< 0.001
Counts_min	15 (10,22.25)	16 (10,24)	21 (13,32)	< 0.001
Counts_mean	31.4 (23.02,41.89)	32.44 (24.12,44.68)	40.1 (30.31,55.29)	< 0.001
Counts_median	30 (21,40)	31 (22,43)	38 (28.25,52)	< 0.001
Counts_sd	6.85 (5.41,9.91)	6.8 (5.41,9.85)	7.97 (6.48,12.44)	< 0.001

### 3.2 The feature extraction and selection

815 radiomics features were extracted from the ROI_C_ and ROI_L_ of each WBS image, respectively, while 2048 deep learning features were also obtained. After the application of a feature selection process, 11 and 12 features were ultimately chosen from the ROI_C_ and ROI_L_ features, respectively, and 15 features were selected from the deep learning features.

### 3.3 Radiomics and DL model evaluation

Following a comparison of the ROI_C_ and ROI_L_ models, the ROI_C_ model achieving superior performance in all five ML algorithms (AUC = 0.806, 0.798, 0.768, 0.800 and 0.758) compared to the ROI_L_ model (AUC = 0.742, 0.747, 0.714, 0.732 and 0.736) in the internal validation cohort. Logistic regression showed the best performance in both the ROI_C_ model (AUC = 0.806; 95% CI = 0.771–0.841) and the DL (AUC = 0.807; 95% CI = 0.772–0.842) in the internal validation cohort(Table 2). There was no statistically significant difference in predicting BM between the ROI_C_ and deep learning models (DeLong test, *p* > 0.05).

**TABLE 2:** The performance of ML models in training and internal validation cohorts.

		Training						Internal validation					
model name	algorithm	AUC	Accuracy	Sensitivity	Specificity	PPV	NPV	AUC	Accuracy	Sensitivity	Specificity	PPV	NPV
ROI_L_ model	Logistic Regression	0.793	0.72	0.76	0.66	0.72	0.71	0.742	0.67	0.74	0.58	0.67	0.67
	KNN	0.802	0.73	0.82	0.64	0.72	0.76	0.747	0.67	0.76	0.58	0.67	0.68
	Random Forest	0.819	0.74	0.81	0.67	0.74	0.75	0.714	0.65	0.73	0.56	0.66	0.65
	SVM	0.775	0.72	0.73	0.70	0.74	0.70	0.732	0.67	0.70	0.64	0.69	0.66
	XGBoost	0.827	0.87	0.74	0.59	0.71	0.80	0.736	0.67	0.79	0.53	0.65	0.69
ROI_C_ model	Logistic Regression	0.823	0.76	0.86	0.65	0.74	0.80	0.806	0.73	0.83	0.61	0.71	0.76
	KNN	0.836	0.75	0.82	0.68	0.74	0.76	0.798	0.73	0.80	0.65	0.72	0.75
	Random Forest	0.899	0.80	0.87	0.73	0.79	0.83	0.768	0.70	0.78	0.62	0.70	0.71
	SVM	0.807	0.76	0.80	0.70	0.76	0.76	0.800	0.73	0.82	0.62	0.71	0.75
	XGBoost	0.872	0.78	0.85	0.69	0.76	0.81	0.758	0.70	0.79	0.61	0.69	0.72
DL model	Logistic Regression	0.821	0.74	0.73	0.76	0.78	0.71	0.807	0.73	0.73	0.74	0.76	0.70
	KNN	0.836	0.75	0.79	0.70	0.75	0.75	0.787	0.75	0.80	0.69	0.74	0.75
	Random Forest	0.867	0.79	0.80	0.77	0.80	0.77	0.788	0.73	0.77	0.69	0.74	0.73
	SVM	0.810	0.74	0.76	0.72	0.75	0.72	0.788	0.72	0.75	0.68	0.73	0.71
	XGBoost	0.867	0.79	0.83	0.75	0.79	0.80	0.789	0.73	0.79	0.66	0.72	0.73

### 3.4 Segmentation reproducibility

All 19 final‐model features showed good inter‐observer reproducibility. The ICCs of the 8 ROIC radiomics features ranged from 0.996 to 0.999, while those of the 11 DL features ranged from 0.943 to 0.998. Individual feature‐level ICCs and 95% confidence intervals are presented in File S2.

### 3.5 RDB‐nomogram validation and explainability

**FIGURE 2:**
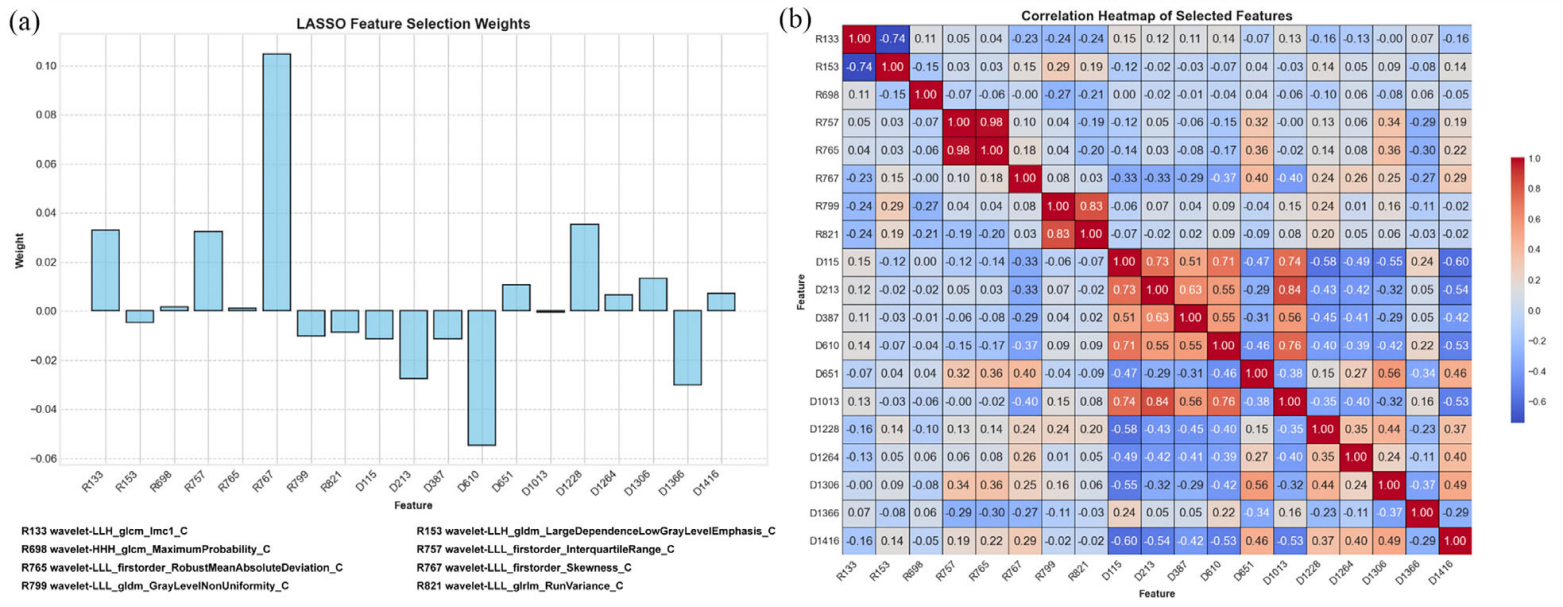
The LASSO coefficient profiles of the final features (a). The heatmap of the correlation between features (b).

**FIGURE 3:**
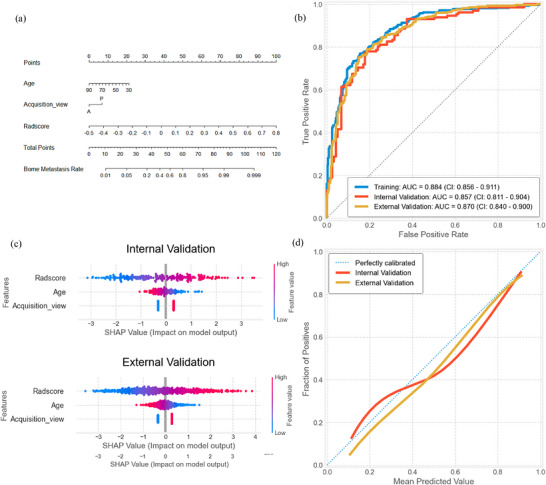
The RDB‐nomogram (a).The ROC curves demonstrate the performance of the RDB‐nomogram in the training, internal validation and external validation cohorts (b). The SHAP plots illustrate the influence of each factors on the model's prediction in the internal validation and external validation cohorts (c). The calibration curve for RDB‐nomogram in the internal validation and external validation cohorts (d).

**FIGURE 4:**
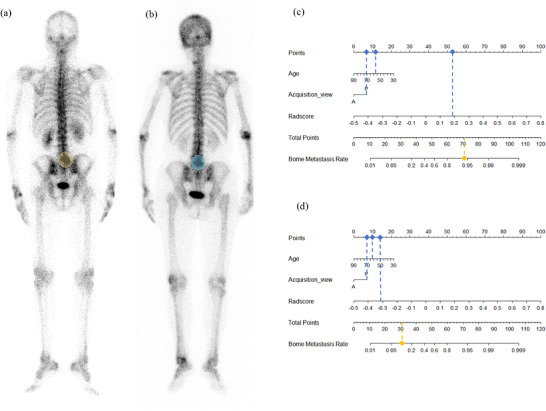
The total point of two NSCLC patients were calculated by using the RDB‐nomogram. a 71‐year‐old woman with BM lesion in posterior view of the WBS image, Radscore = 0.196 (a,c). a 62‐year‐old women with vertebral degeneration with posterior view of the WBS image, Radscore = −0.319 (b,d).

After the re‐selection of the ROI_C_ and DL features, the RDB model was developed based on selected 8 ROI_C_ and 11 DL features using the logistic regression algorithm(Figure 2; File S3). The RDB rad‐score of each patient was generated (File S4). The univariate and multivariable logistic regression results indicated that age, acquisition view, and Rad‐score were independent factors for the prediction of BM (Table 3). Accordingly, an RDB‐nomogram was constructed based on these factors and exhibited an AUC value of 0.857 (95% CI: 0.811–0.904) and 0.870 (95% CI: 0.840–0.900) in the internal and external validation cohorts (Figure 3a,b). The Hosmer‐Lemeshow test demonstrated excellent calibration of the nomogram in the training (*p* = 0.619), internal validation (*p* = 0.979), and external validation cohorts (*p* = 0.667). The DCA also showed RDB‐nomogram was a reliable tool for predicting BM in NSCLC (File S5). The calibration curve is presented in Figure 3d. The SHAP method was employed to demonstrate the impact of each factor on the model's prediction. A positive SHAP value shows an elevated likelihood of a specific outcome, whereas a negative value indicates a diminished probability. The RDB Rad‐score is the most significant factor in both internal and external validation cohorts (Figure 3c). An example of the nomogram in use is shown in Figure 4.

**TABLE 3:** Univariable and multivariable logistic regression analysis.

	Univariable analysis		Multivariable analysis	
Variable	OR [95% CI]	*p* value	OR [95% CI]	*p* value
Age	0.958(0.941–0.975)	< 0.001	0.948(0.925–0.973)	< 0.001
Gender	1.170(0.835–1.640)	0.361	–	
Lesion voxel number	1.000(1.000–1.002)	0.160	–	
Lesion size(cm2)	1.192(0.933–1.524)	0.160	–	
Counts_max	1.031(1.023–1.039)	< 0.001	0.989(0.929–1.052)	0.721
Counts_min	1.044(1.027–1.061)	< 0.001	1.01(0.925–1.103)	0.821
Counts_mean	1.042(1.030–1.054)	< 0.001	1.036(0.708–1.515)	0.855
Counts_median	1.040(1.028–1.053)	< 0.001	0.972(0.708–1.336)	0.863
Counts_sd	1.295(1.214–1.380)	< 0.001	1.121(0.897–1.400)	0.315
location	1.171(1.005–1.365)	0.043	0.808(0.641–1.019)	0.072
Acquisition view	1.403(1.011–1.946)	0.043	1.772(1.085–2.893)	0.022
Radscore	7588.030(1819.069–31652.570)	< 0.001	7920.792(1394.788–44980.980)	< 0.001

### 3.6 RDB‐nomogram for overall survival analysis

**FIGURE 5:**
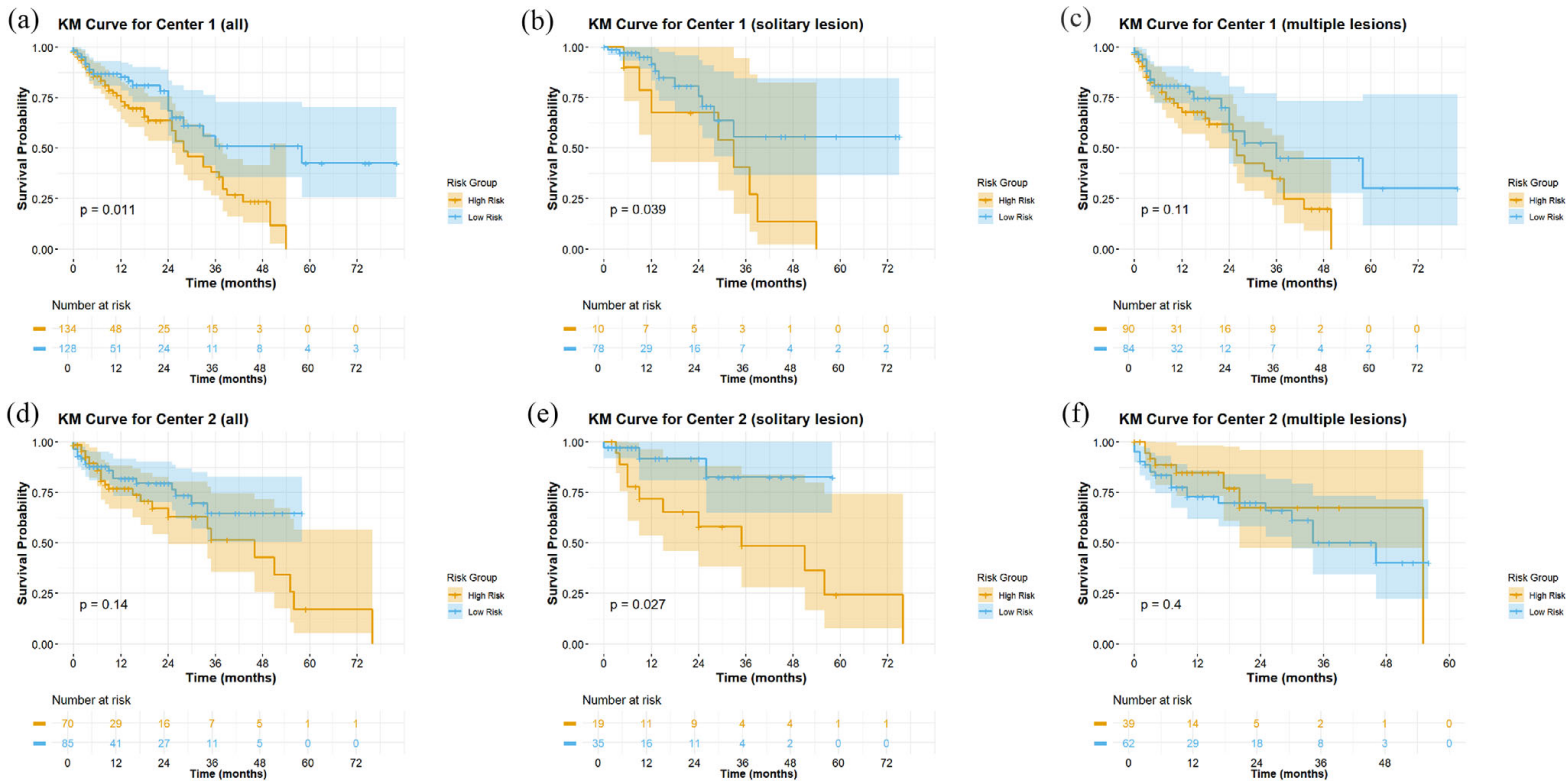
Kaplan‐Meier survival curves stratified by risk groups based on RDB‐nomogram total points. (a–c) all patients, solitary lesion subgroup and multiple lesions group in Center 1; (d–f) all patients, solitary lesion subgroup and multiple lesions group in Center 2.

We evaluated the predictive value of the RDB‐nomogram using available follow‐up data from patients with BM. In Center 1, confirmable OS information was available for 262 of 441 BM patients (88 solitary and 174 multiple BM lesions); the average follow‐up period was 13.53 months (1–82 months), with 76 confirmed deaths. In Center 2, confirmable OS information was available for 155 of 279 BM patients (54 solitary and 101 multiple BM lesions); the average follow‐up period was 16.40 months (1–76 months), with 43 death endpoints. Patients without reliable vital status or survival time were not included in the OS analysis. Kaplan‐Meier curve analysis confirmed these findings, showing significant differences in OS between high‐ and low‐risk groups across both cohorts. In Center 1, the OS difference was significant overall (*p* = 0.011), with the solitary lesion subgroup demonstrating a more dramatic contrast (15.2 vs. 24.8 months, *p* = 0.039) compared to the multiple lesions subgroup, which did not reach statistical significance (*p* = 0.11). In Center 2, the overall survival difference was also significant (*p* = 0.037), with the solitary lesion subgroup exhibiting a stronger predictive performance (13.1 vs. 28.6 months, *p* = 0.027), while the multiple lesions subgroup showed no significant difference (*p* = 0.4) (Figure 5a–f).

### 3.7 Software deployment and reproducibility

**FIGURE 6:**
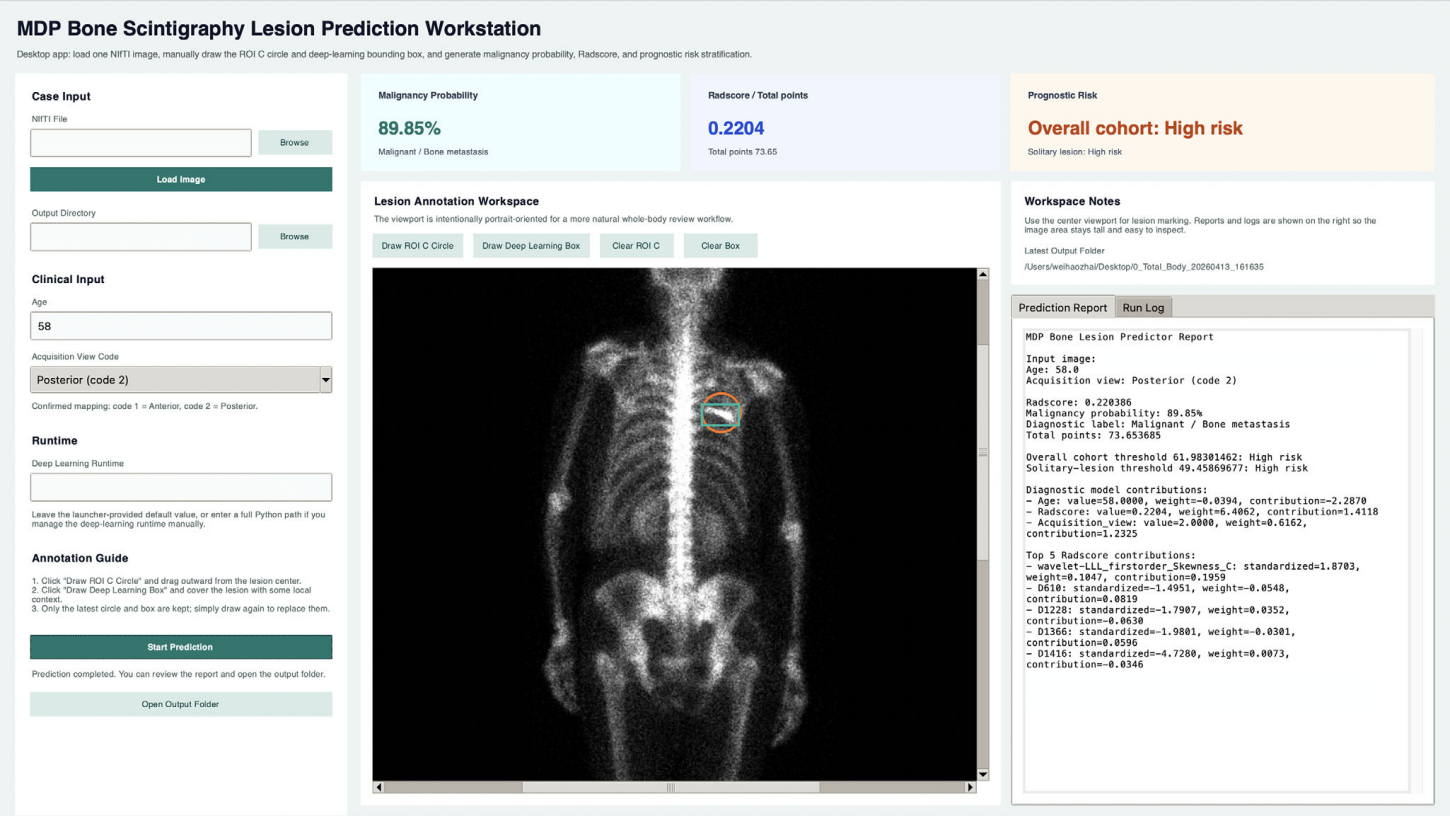
Example of the desktop implementation. The software provides a local graphical user interface for lesion‐level analysis on planar whole‐body bone scintigraphy. Users first load a single WBS image in NIfTI format and enter the required clinical information. A circle‐shaped ROI C is then manually drawn for radiomics feature extraction, and a rectangular bounding box is placed over the lesion for deep‐learning feature extraction. After annotation, the program automatically computes the Rad‐score, lesion malignancy probability, and nomogram‐based prognostic risk, and generates a prediction report together with the corresponding output files.

Based on the finalized model coefficients and feature‐selection results, we completed a desktop software deployment of the RDB‐nomogram (Figure 6). The released implementation preserves the final 8 radiomics features, 11 deep learning features, and 2 clinical factors, and provides lesion‐level malignancy probability, Rad‐score, and nomogram‐based prognostic risk after manual annotation of a single WBS image. The application is not intended for research only, supporting the prospective multicenter validation, broader scanner/protocol testing, and regulatory assessment.

## 4 DISCUSSION

This study specifically addresses the challenge of differentiating osteoblastic high MDP‐avid BM lesions in NSCLC patients, we gradually developed our RDB‐nomogram from radiomics, DL, and clinical factors. Meanwhile, using SHAP values and SHAP plots, we can describe the most important features for predicting BM in NSCLC patients. The RDB‐nomogram demonstrated robust diagnostic performance in internal and external validation and showed exploratory prognostic potential, especially in patients with solitary BM lesions.

While SPECT/CT and CT offers higher resolution and anatomical information for identifying early BM, the clinical utilization of WBS is more prevalent owing to the examination's cost and the broad availability of equipment. Previous studies indicate that gamma cameras and SPECT systems, which are incapable of acquiring CT images, still contribute 40% of clinical applications, particularly in developing nations.^23^, ^24^ Moreover, WBS has a higher sensitivity than CT and MR and offers a whole body vision for screening. Therefore, we chose WBS images to establish a model suitable for all devices, especially for the MDP‐avid area. To the best of our knowledge, few studies investigated the radiomics model based on planar WBS images. A study by Wang et al. demonstrated that a Random Forest model based on clinical features and radiomics features derived from bone scintigraphy can effectively predict recurrence‐free survival in patients with local or locally advanced prostate cancer after initial treatment. This study attempts to further combine radiomics with DL to establish a diagnostic model.^25^ In prior radiomics studies, segmentation typically relied on the lesion's boundaries. The superior performance of ROIC compared with ROIL may be explained by the inclusion of perilesional information. ROIC captures not only the high‐uptake lesion core but also the lesion‐to‐background transition zone and adjacent bone remodeling, which may contain discriminative information for differentiating BM from degenerative or inflammatory uptake. By contrast, ROIL is more restricted to the visible high‐uptake boundary and may be more sensitive to edge uncertainty and partial‐volume effects on planar WBS. This conclusion is consistent with some previous radiomics studies based on peritumoral tissue features.^26^, ^27^ The eight radiomics features chosen in our model were all derived from wavelet images, likely due to its efficacy in noise reduction and enhancement of image details compared to the original image, thereby facilitating the extraction of more precise and valuable image features. Previous radiomics studies of 99mTc‐MDP bone scan were all based on SPECT/CT images and several models have been published for the diagnosis of bone lesions in recent years. A researcher investigated the application of SPECT/CT radiomics features to differentiate between chondromas and grade I chondrosarcomas (atypical chondromas, ACTs) in long bones, discovering that increased radiomics features were significantly associated with the diagnosis of ACTs.^28^ Wang et al. developed a SPECT/CT‐based model that combines clinical and radiomics information (7 CT+ 1 SPECT) to predict the differentiation between BM and benign bone diseases in patients with malignant tumors and obtained the highest AUC of 0.879 in the testing group and 0.853 in the validation group.^29^ This model is established based on multimodal radiomics features, predominantly comprised CT features. Our model, which is based solely on WBS images, has achieved comparable performance. A radiomics model integrating 18 SPECT and CT features developed by Jin et al. showed superior classification performance (AUC = 0.926) in differentiating vertebral BM compared to human experts and individual CT and SPECT models.^30^ Wang et al. constructed a radiomics‐clinical model using Rad‐score and 2 clinical factors for lung cancer patients with support vector machines (SVM) to identify BM and received a higher AUC of 0.925 in the testing group.^31^ In a study by Wang et al., they proposed and validated a prediction model based on radiomics features and 3 clinical features with 4 different ML algorithms for predicting BM in prostate cancer patients.^32^ The logistic regression model showed the highest AUC values (0.984 and 0.916) in the training and test groups, surpassing human expert evaluations. Some of these models achieved higher AUC values than ours, but they often involved smaller cohorts, single‐center or limited validation designs, or multimodal SPECT/CT features. In contrast, our RDB‐nomogram used a larger two‐center cohort, an external validation set, planar WBS images alone, integration of radiomics and DL features, and a software implementation.

Previous studies have shown that the one‐year survival rate for NSCLC patients with BM ranges from 40% to 50%.^33^, ^34^, ^35^ To further explore the added value of our RDB‐nomogram, we conducted an KM analysis in two centers. Significant survival differences were observed in all BM patients and, more consistently, in solitary‐lesion subgroups. By contrast, the multiple‐lesion subgroups did not show significant OS separation. A plausible explanation is that our segmentation strategy used a single dominant lesion per patient. Although this approach is analytically consistent for lesion‐level diagnosis, it may not capture whole‐body metastatic burden, inter‐lesion heterogeneity, treatment response, or systemic disease severity in multifocal BM. Therefore, the negative multiple‐lesion survival results are consistent with the selection‐bias limitation of single‐lesion analysis and suggest that future prognostic models for multiple BM should incorporate patient‐level lesion burden or whole‐body quantitative features.

An additional strength of this study is that the final pipeline was translated into an openly accessible software tool rather than remaining limited to analytic notebooks. By releasing a local desktop implementation with a standardized annotation workflow and automated deployment scripts we sought to lower the technical barrier for external validation and facilitate use in centers where planar WBS remains the primary screening modality. Nevertheless, the current software should still be regarded as a research‐use tool, and prospective validation, broader hardware adaptation, and regulatory assessment are required before routine clinical adoption.

 Our study has several limitations. First, the study cohorts included patients with multiple BM. To maintain analytical consistency, we selected the most prominent lesion for analysis, assuming it to be the dominant metastatic focus. However, this single‐lesion strategy may overlook biological heterogeneity and whole‐body disease burden in multifocal BM, introduce selection bias, and partly explain the weaker prognostic performance in multiple‐lesion subgroups. Future prognostic studies should incorporate patient‐level lesion burden or whole‐body quantitative features. Second, histological confirmation of BM was unavailable for all patients, necessitating reliance on imaging follow‐up—a pragmatic but imperfect criterion. Third, OS follow‐up was only for a subset of BM patients and the duration was relatively short; therefore, the survival analysis should be interpreted as exploratory and may be affected by follow‐up selection bias. Finally, future prospective studies should incorporate additional clinical factors, especially those closely associated with BM, to further evaluate the efficacy of the nomogram.

## 5 CONCLUSIONS

In summary, we developed an RDB‐nomogram that integrates the clinical factors, radiomics, and DL features that help to predict MDP‐avid BM in NSCLC patients for precise therapeutic strategies. By focusing on osteoblastic lesions and demonstrating its prognostic value, our RDB‐nomogram may function as an efficient diagnostic and screening tool for non‐invasive BM prediction and in facilitating the formulation of an individualized therapeutic strategy. The research‐use‐only desktop implementation provides a practical pathway for reproducibility and prospective multicenter broader external validation.

## AUTHOR CONTRIBUTIONS

The following is the contributions of all authors:

(First Author)Weihao Zhai: Conceptualization, Methodology, Validation, Formal analysis, Visualization, Writing—Original Draft. Xiaolin Li: Investigation, Data Curation, Conceptualization. Qian Zhou: Data Curation. Taohu Zhou: Data Curation, Validation. Xiaoqing Lin: Data Curation. Xiuxiu Zhou: Data Curation, Methodology. Xin'ang Jiang: Data Curation. Ziwei Zhang: Data Curation. Qianxi Jin: Data Curation. Shiyuan Liu: Project administration. (Corresponding Author)Li Fan: Writing ‐review & editing.

## CONFLICT OF INTEREST STATEMENT

The authors of this manuscript declare no relationships with any companies, whose products or services may be related to the subject matter of the article.

## DATA AVAILABILITY STATAEMENT

The data and software supporting this work are available from the authors on reasonable request during peer review. The code of desktop software deployment of the RDB‐nomogram is published at: https://github.com/AI‐Lung/Bone‐MDP‐Prediction.

## ETHICS STATEMENT

The Research Ethics Committee of the Institutional Review Boards of the 2 centers (Second Affiliated Hospital of Naval Medical University and Huadong Hospital) approved this retrospective study (No.2024SL135).

## CONSENT TO PARTICIPATE

The Research Ethics Committee of the Institutional Review Boards of the 2 centers waived the informed consent.

## Supporting information



Supporting Information
