# Supplementary material for: An interpretable radiomics–deep learning nomogram from whole‐body bone scintigraphy for MDP‐avid bone metastasis prediction in NSCLC
Source: J Appl Clin Med Phys. 2026 Aug 3;27(8):e70730. doi: 10.1002/acm2.70730 (PMC13433491; doi:10.1002/acm2.70730)
Supplement: Supplementary file 1 [file ACM2-27-e70730-s001.docx]

**Supplementary Files**

An Interpretable Radiomics–Deep Learning Nomogram From Whole-Body Bone Scintigraphy for MDP-Avid Bone Metastasis Prediction in NSCLC

Supplementary File 1: Machine Learning model construction

Supplementary File 2:Inter-observer reproducibility of the features

Supplementary File 3: Feature Selection (ROI_L_,ROI_C_,Deep)

Supplementary File 4: Calculation of RDB rad-score

Supplementary File 5:The decision curve of RDB-nomogram

**Supplementary File 1: Machine Learning model construction**

To select the best performance of the model. We are using the 5 machines learning algorithms. The patients were divided into training and internal validation cohort randomly with a 7:3 ratio in center 1. To identify the best model hyperparameters, grid search technique was applied.

1. Logistic Regression

Logistic Regression is a statistical method for classifying observations into two or more discrete categories. It uses a logistic function to model the probability of a certain class as a function of the independent variables. Logistic Regression is often used in medical research, social sciences, and marketing to predict binary outcomes.

2. K-Nearest Neighbors (KNN)

Nearest Neighbors is a simple yet effective supervised learning algorithm for classification and regression. It assigns the class label or regression value of an unknown observation based on the most common class or mean value among its K nearest neighbors in the training data.

3. Random Forest

Random Forest is an ensemble method that constructs a multitude of decision trees during training and outputs the class that is the mode of the classes (classification) or mean prediction (regression) of the individual trees. Random Forest introduces additional randomness in the construction of the trees to decorrelate the trees and improve performance.

4. Support Vector Machine (SVM)

Support Vector Machine is a supervised learning algorithm used for data classification. It works by finding a hyperplane in a high-dimensional space that best separates the data points into their respective categories. SVMs are often used for data classification problems, including text and image classification.

5. XGBoost

XGBoost is an optimized distributed gradient boosting library that is designed to be efficient, flexible, and portable. It implements machine learning algorithms under the gradient boosting framework and builds on decision tree structures. XGBoost offers parallel tree boosting, which enables it to handle large-scale datasets efficiently and accurately. It also includes linear model solvers and tree learning algorithms, making it a powerful tool for structured and tabular datasets.

**Supplementary File 2:Inter-observer reproducibility of the features**

| Feature group | Feature | ICC | 95% CI |
| --- | --- | --- | --- |
| ROI_C | wavelet-LLH_glcm_Imc1_C | 0.997 | 0.993–0.999 |
| ROI_C | wavelet-LLH_gldm_LargeDependenceLowGrayLevelEmphasis_C | 0.999 | 0.998–0.999 |
| ROI_C | wavelet-HHH_glcm_MaximumProbability_C | 0.999 | 0.999–0.999 |
| ROI_C | wavelet-LLL_firstorder_InterquartileRange_C | 0.997 | 0.968–0.999 |
| ROI_C | wavelet-LLL_firstorder_RobustMeanAbsoluteDeviation_C | 0.996 | 0.970–0.999 |
| ROI_C | wavelet-LLL_firstorder_Skewness_C | 0.997 | 0.987–0.999 |
| ROI_C | wavelet-LLL_gldm_GrayLevelNonUniformity_C | 0.999 | 0.999–0.999 |
| ROI_C | wavelet-LLL_glrlm_RunVariance_C | 0.999 | 0.999–0.999 |
| DL | D115 | 0.986 | 0.954–0.998 |
| DL | D213 | 0.986 | 0.969–0.994 |
| DL | D387 | 0.976 | 0.884–0.996 |
| DL | D610 | 0.993 | 0.987–0.998 |
| DL | D651 | 0.985 | 0.965–0.994 |
| DL | D1013 | 0.976 | 0.934–0.994 |
| DL | D1228 | 0.984 | 0.961–0.996 |
| DL | D1264 | 0.998 | 0.996–0.999 |
| DL | D1306 | 0.990 | 0.983–0.996 |
| DL | D1366 | 0.992 | 0.980–0.998 |
| DL | D1416 | 0.943 | 0.897–0.991 |

**Supplementary File 3: Feature Selection (ROIL,ROIC,Deep)**

1. ROI_L_ Feature

2. ROI_C_ Feature

3. Deep Feature

**Supplementary File 4: Calculation of RDB rad-score**

Rad-score=0.0330*wavelet-LLH_glcm_Imc1_C-0.0047*wavelet-LLH_gldm_LargeDependenceLowGrayLevelEmphasis_C+0.0016*wavelet-HHH_glcm_MaximumProbability_C+0.032*wavelet-LLL_firstorder_InterquartileRange_C+0.0011*wavelet-LLL_firstorder_RobustMeanAbsoluteDeviation_C+0.1047*wavelet-LLL_firstorder_Skewness_C-0.0102*wavelet-LLL_gldm_GrayLevelNonUniformity_C-0.0087*wavelet-LLL_glrlm_RunVariance_C-0.0112*D115-0.0274*D213-0.0113*D387-0.0548*D610+0.0105*D651-0.0003*D1013+0.0352*D1228+0.0067*D1264+0.0133*D1306-0.0301*D1366+0.0073*D1416

**Supplementary File 5:The decision curve of RDB-nomogram**
